# Evolutionary Dynamics of Host Organs for Microbial Symbiosis in Tortoise Leaf Beetles (Coleoptera: Chrysomelidae: Cassidinae)

**DOI:** 10.1128/mbio.03691-21

**Published:** 2022-01-25

**Authors:** Kayoko Fukumori, Kohei Oguchi, Hiroshi Ikeda, Tadashi Shinohara, Masahiko Tanahashi, Minoru Moriyama, Ryuichi Koga, Takema Fukatsu

**Affiliations:** a National Institute of Advanced Industrial Science and Technology (AIST), Tsukuba, Japan; b Biodiversity Division, National Institute for Environmental Studies, Tsukuba, Japan; c Faculty of Agriculture and Life Science, Hirosaki University, Hirosaki, Japan; d Graduate School of Human Development and Environment, Kobe University, Kobe, Japan; e Department of Applied Chemistry, National Chiao Tung University, Hsinchu, Taiwan; f Department of Biological Sciences, Graduate School of Science, University of Tokyo, Tokyo, Japan; g Graduate School of Life and Environmental Sciences, University of Tsukuba, Tsukuba, Japan; University of Hawaii at Manoa

**Keywords:** tortoise leaf beetle, Cassidinae, symbiotic bacteria, *Stammera capleta*, symbiotic organ, symbiont loss

## Abstract

Diverse insects host specific microbial symbionts that play important roles for their growth, survival, and reproduction. They often develop specialized symbiotic organs for harboring the microbial partners. While such intimate associations tend to be stably maintained over evolutionary time, the microbial symbionts may have been lost or replaced occasionally. How symbiont acquisitions, replacements, and losses are linked to the development of the host’s symbiotic organs is an important but poorly understood aspect of microbial symbioses. Cassidine leaf beetles are associated with a specific gammaproteobacterial lineage, *Stammera*, whose reduced genome is streamlined for producing pectin-degrading enzymes to assist the host’s digestion of food plants. We investigated the symbiotic system of 24 Japanese cassidine species and found that (i) most species harbored *Stammera* within paired symbiotic organs located at the foregut-midgut junction, (ii) the host phylogeny was largely congruent with the symbiont phylogeny, indicating stable host-symbiont association over evolutionary time, (iii) meanwhile, the symbiont was not detected in three distinct host lineages, uncovering recurrent losses of the ancient microbial mutualist, (iv) the symbiotic organs were vestigial but present in the symbiont-free lineages, indicating evolutionary persistence of the symbiotic organs even in the absence of the symbiont, and (v) the number of the symbiotic organs was polymorphic among the cassidine species, either two or four, unveiling a dynamic evolution of the host organs for symbiosis. These findings are discussed as to what molecular mechanisms and evolutionary trajectories underpin the recurrent symbiont losses and the morphogenesis of the symbiotic organs in the herbivorous insect group.

## INTRODUCTION

Insects represent the biodiversity of the terrestrial ecosystem, wherein microbial symbioses are omnipresent and play important biological roles (1, 2). Some microbial symbionts are facultative associates that affect their hosts either negatively or positively, often in a condition-dependent manner (3, 4). Other microbial symbionts are obligatory partners that are indispensable for normal growth, survival, and reproduction of their hosts by supplying essential nutrients (5, 6), helping food digestion (7, 8), or conferring defense against natural enemies (9, 10). Many, if not all, of these intimate microbial symbioses entail the development of specialized cells, tissues, and organs of the host insects for hosting microbial symbionts (11–13). In many intimate gut microbial symbioses, specific symbionts are harbored extracellularly in a specialized gut region with tubular or pouch-like outgrowths, called midgut crypts or gastric ceca, in many stinkbugs, some beetles, fruit flies, and others (14–23). In many endocellular microbial symbioses, specific symbionts are hosted within the cytoplasm of specialized cells called bacteriocytes, which may constitute distinct organs called bacteriomes, in aphids and other hemipterans, lygaeid stinkbugs, weevils, grain beetles, tsetse flies, ants, cockroaches, and others (18, 24–33). In addition, it should be noted that there are a variety of intermediate symbiotic configurations: for example, in anobiid beetles and leaf beetles, the symbiotic bacteria are located not only in the cavity of gastric ceca extracellularly but also within the cecal epithelial cells endocellularly (14, 16, 17, 22), and in ants and tsetse flies, the bacteriocytes are localized to or embedded in a specific region of the midgut epithelium (24, 27). The origin and evolution of these diverse insect organs for microbial symbioses, so-called “symbiotic organs,” constitute a challenging problem in evolutionary developmental biology.

Such obligatory and beneficial microbial symbionts hosted in specialized symbiotic organs tend to be conserved in the evolutionary course of the host insects because of the mutualistic nature of the host-symbiont associations. Meanwhile, such mutualistic symbionts have been lost or replaced occasionally (34, 35). For example, in weevils (Coleoptera: Curculionidae), while diverse weevil groups are commonly associated with the endosymbiotic bacterium *Nardonella* that provides its hosts with tyrosine for cuticle hardening (31), some weevil lineages have either lost the ancient symbiotic association or renewed the association with new partners like *Sodalis* in grain weevils and *Curculioniphilus* in acorn weevils (36–38). In aphids (Hemiptera: Aphidoidea), while most species are associated with the endosymbiotic bacterium *Buchnera* that nutritionally supports its hosts by the provisioning of essential amino acids (5, 6), the ancient endosymbiont has been replaced by other bacteria or fungi in several aphid lineages and is completely lost in the sister taxon Phylloxeridae (39–42). In heteropteran bugs (Hemiptera: Heteroptera), while the majority of plant-sucking groups are associated with diverse symbiotic bacteria in the midgut symbiotic organ extracellularly, the microbial associations have been lost in predacious bug lineages, and in the family Lygaeidae, strikingly, the bacteriomes have newly evolved repeatedly to establish novel endosymbiotic associations (32, 43–45). How such symbiont losses and replacements affect the formation, development, and evolution of the symbiotic organs of the host insects is of great interest.

Tortoise leaf beetles (Coleoptera: Chrysomelidae) comprise the second largest leaf beetle subfamily, Cassidinae (46). Recent studies unveiled that tortoise leaf beetles possess the symbiotic bacterium “*Candidatus* Stammera capleta” (hereafter designated *Stammera*), whose genome has been reduced to less than 0.3 Mb and specialized for production of pectinases, thereby assisting the host’s digestion of plant cell wall and supporting the host’s normal growth and survival (22, 47). An early histological study described paired symbiotic organs located at an anterior part of the alimentary tract of both females and males and also a pair of tubular organs for vertical symbiont transmission connected to the female’s reproductive system (16). These symbiotic configurations were confirmed using modern molecular and histological techniques on the thistle tortoise leaf beetle, Cassida rubiginosa (22). Meanwhile, the early study (16) mentioned that several cassidine species (Cassida nebulosa and Cassida flaveola) were devoid of the symbiotic bacteria and the symbiotic organs, suggesting loss(es) of the presumably essential microbial symbiosis despite the host-symbiont conservation and cospeciation (47). Since the pioneer work in the 1930s, no study has been conducted on the evolutionary dynamics of the symbiotic system of cassidine leaf beetles.

In this study, we investigated the majority of Japanese cassidine leaf beetles representing 6 genera and 24 species, which uncovered dynamic evolutionary processes entailing recurrent symbiont losses and morphological changes of the symbiotic organs.

## RESULTS

### *Stammera* localization in specialized symbiotic structures throughout the life stages of a cassidine leaf beetle.

**FIG 1:**
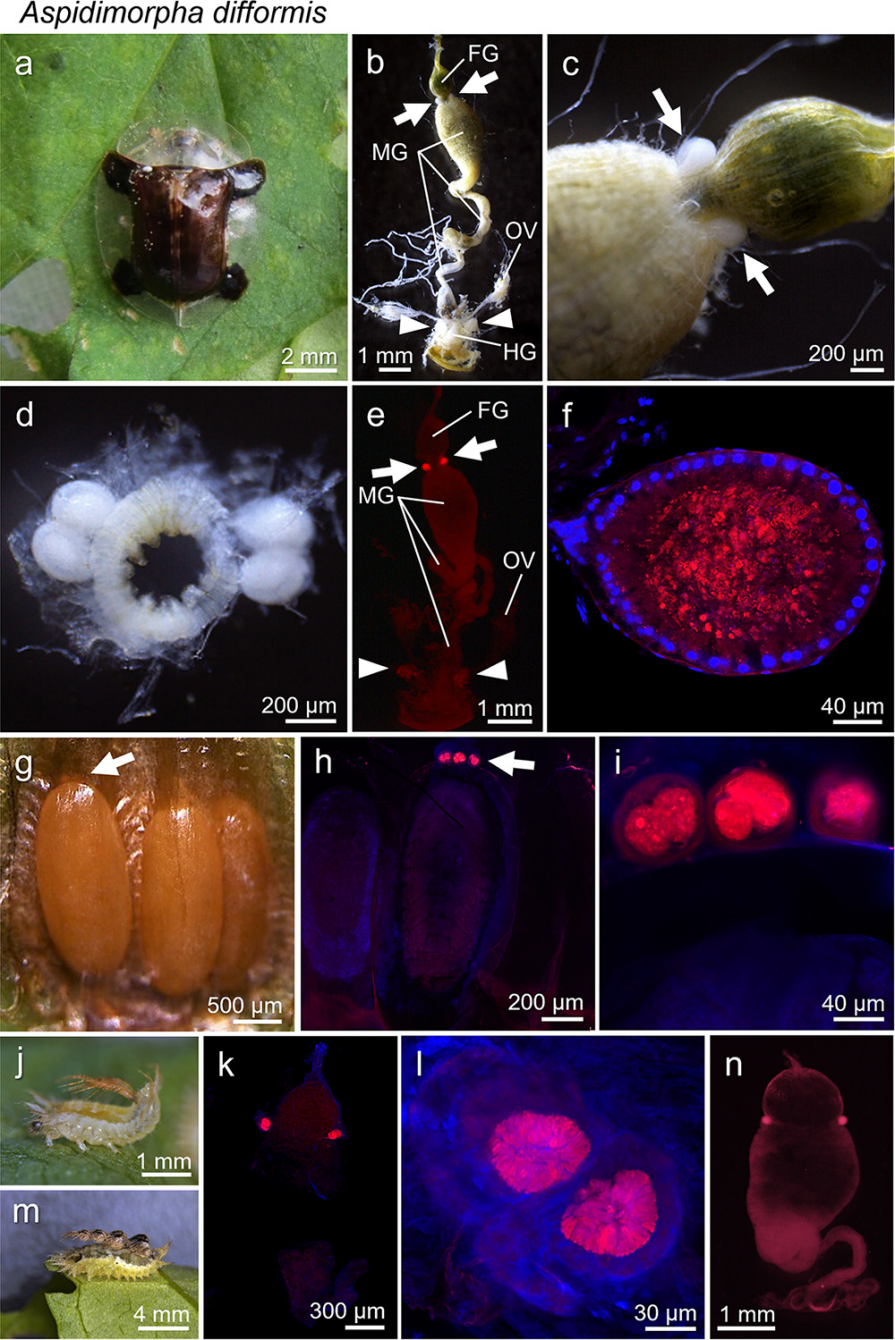
Symbiotic system of *Aspidimorpha difformis*. (a to f) Adult insects. (a) An adult. (b) A dissected alimentary tract of an adult female. Arrows show the gut-associated symbiotic organs, and arrowheads show the female-specific genital accessory organs. Abbreviations: FG, foregut; HG, hindgut; MG, midgut; OV, ovary. (c) Symbiotic organs located on both sides of the foregut-midgut junction (arrows). (d) An excised foregut-midgut junction on which four symbiotic organs are seen. (e) Symbiont localization visualized by whole-mount FISH. Red symbiont signals are seen in the gut-associated symbiotic organs (arrows) and the ovary-associated transmission organs (arrowheads). (f) An enlarged FISH image of the symbiotic organ. Red symbiont signals are detected in the inner cavity of the organ. Nuclei of the monolayer epithelial cells of the organ are counterstained in blue. (g to i) Eggs. (g) Eggs arranged in the chitinous egg case. An arrow indicates the symbiont caplets on the anterior pole of an egg. (h) Whole-mount FISH visualizes symbiont signals localized to the caplets (arrow). (i) An enlarged FISH image of the caplets. (j to l) First-instar larvae. (j) A first-instar larva whose abdomen is bent upwards with a cuticular appendage presumably for camouflage. (k) Whole-mount FISH of the dissected alimentary tract. Red symbiont signals are detected in four symbiotic organs which are paired on both sides of the foregut-midgut junction. (l) An enlarged FISH image of the paired symbiotic organs. (m and n) Fifth-instar larvae. (m) A fifth-instar larva whose abdomen is bent upwards with four connected shed skins decorated with feces on the back. (n) Whole-mount FISH of the dissected alimentary tract. Red symbiont signals are detected in four symbiotic organs as in first-instar larvae. For FISH, the general bacterial probe Al555-EUB338 (e) and the *Stammera*-specific probe Al555-Tor971 (f, h, i, k, l, and n) were used.

First, we investigated the specialized symbiotic structures and *Stammera* localization throughout the life stages of a cassidine species, Aspidimorpha difformis. In adult insects (Fig. 1a), gut-associated symbiotic organs were found on both sides of the foregut-midgut junction (Fig. 1b and c). On each side, two oval organs were connected to the gut via a thin duct, and four symbiotic organs were present in total (Fig. 1d). Fluorescence *in situ* hybridization (FISH) detected *Stammera* cells in the inner cavity of the symbiotic organs (Fig. 1e and f). In addition, specifically in adult females, a pair of genital accessory organs, which exhibited FISH signals of the symbiont cells, were found in association with the ovaries (Fig. 1b), representing the specialized organs for vertical symbiont transmission to the offspring (Fig. 1e). On the anterior pole of the eggs that were encased in chitinous egg cases, special structures called “caplets” were observed (Fig. 1g). The caplets exhibited FISH signals of the symbiont cells inside (Fig. 1h and i), which are to be ingested by newborn larvae upon hatching to establish vertical symbiont transmission. Throughout the larval stages, a structural configuration for symbiosis similar to that of adults, namely, four symbiotic organs located at the foregut-midgut junction, was observed (Fig. 1j to n). These results generally agreed with the previous reports on the symbiotic system observed in *C. rubiginosa* and other cassidine species (16, 22).

### Pleomorphic *Stammera* cells in gut-associated symbiotic organs and female-specific genital accessory organs of cassidine leaf beetles.

**FIG 2:**
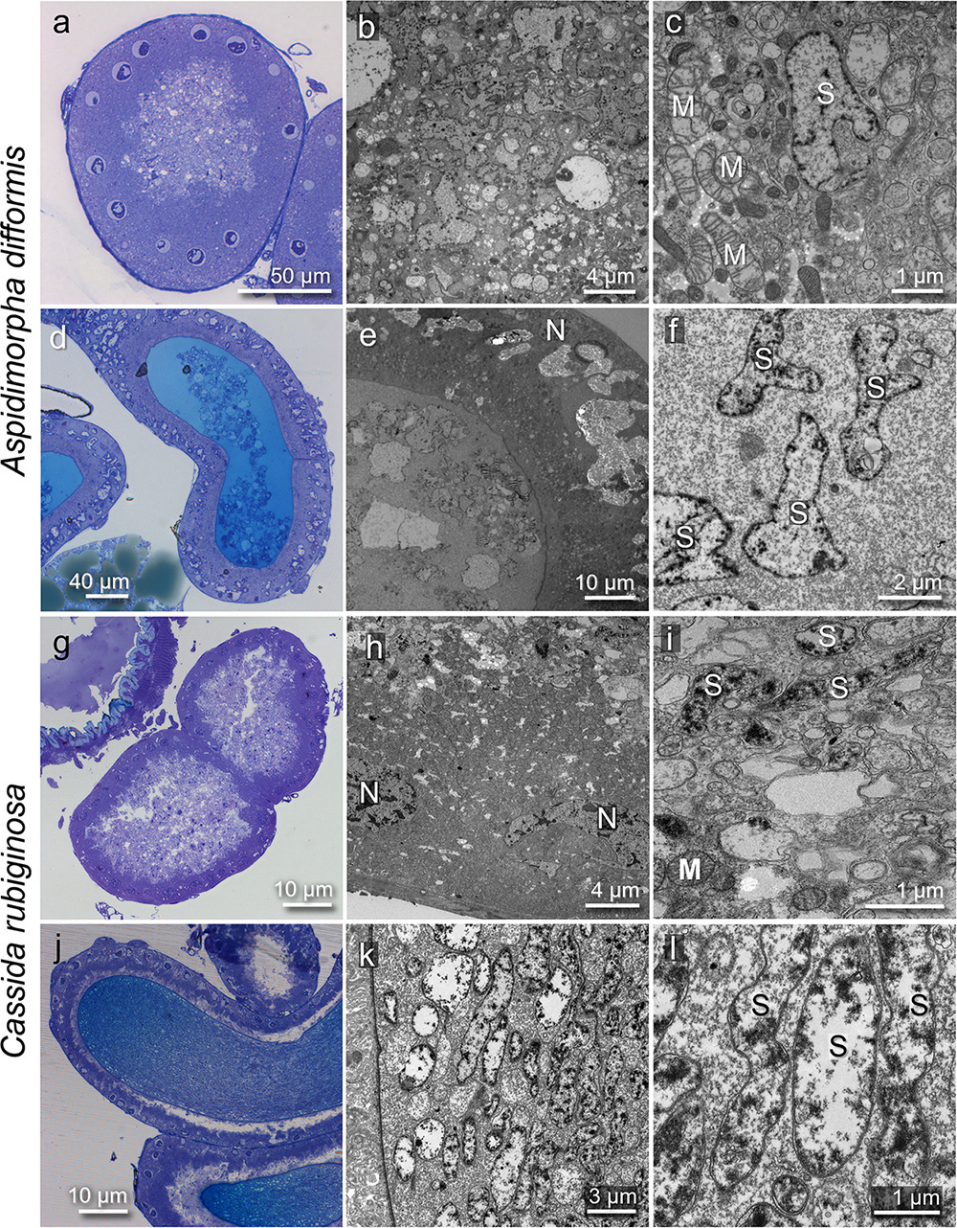
Fine structure of the gut-associated symbiotic organs and the ovary-associated transmission organs of cassidine leaf beetles. (a to f) *Aspidimorpha difformis*. (g to l) *Cassida rubiginosa*. (a to c and g to i) Gut-associated symbiotic organs. (d to f and j to l) Ovary-associated transmission organs. (a, d, g, and j) Light microscopic images of semiultrathin tissue sections stained with toluidine blue. (b, e, h, and k) Transmission electron microscopy (TEM) images of ultrathin tissue sections in which pleomorphic/deformed symbiont cells are seen. (c, f, i, and l) Enlarged TEM images. Abbreviations: M, mitochondrion; N, nucleus; S, symbiont.

We next examined the fine structure of the gut-associated symbiotic organs and the female-specific genital accessory organs of *A. difformis* by using light and electron microscopy. Pleomorphic symbiont cells degenerative in shape were observed in the inner cavity of the symbiotic organ (Fig. 2a and b). While most of the symbiont cells were seen extracellularly, some symbiont cells were found beside mitochondria (Fig. 2c), indicating that *Stammera* cells exist not only extracellularly but also intracellularly, as depicted in previous studies (16, 22). In the female-specific genital accessory organs, in contrast, pleomorphic symbiont cells were exclusively found in the inner cavity extracellularly (Fig. 2d to f). Similar ultrastructural features of the symbiont cells were observed in the symbiotic organs and genital accessory organs of *C. rubiginosa* (Fig. 2g to l). These observations were concordant with the previous reports that *Stammera* is an extracellular symbiont with an extremely reduced genome and pleomorphic cytology (22, 47).

### Host-symbiont cospeciation in cassidine leaf beetles.

**FIG 3:**
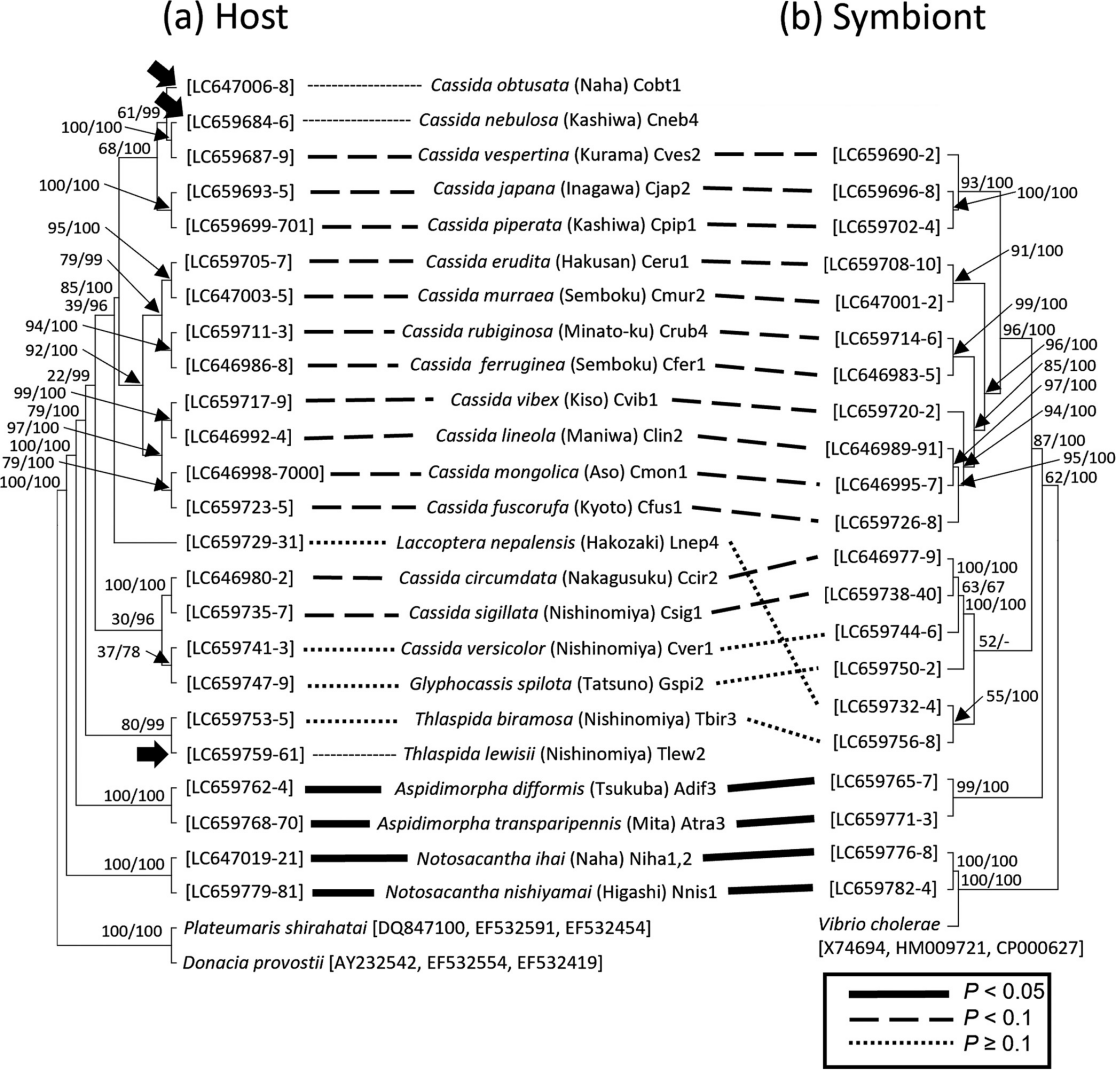
Phylogenetic comparison between cassidine leaf beetles and their *Stammera* symbionts. (a) Host phylogeny inferred by the maximum-likelihood method based on 785, 543, and 804 aligned nucleotide sites of the mitochondrial cytochrome oxidase I gene, the mitochondrial 16S rRNA gene, and the nuclear 28S rRNA gene, respectively. (b) Symbiont phylogeny inferred by the maximum-likelihood method based on 1,569, 903, and 1,443 aligned nucleotide sites of 16S rRNA, *gyrB*, and *groEL* genes, respectively. Note that *groEL* of *C. murraea* is treated as missing data. The bootstrap probability value with 1,000 resamplings of the maximum-likelihood analysis and the posterior probability value of Bayesian analysis are shown at each node. Symbiont losses are indicated on the host phylogeny by thick arrows. Host-symbiont relationships are depicted by dashed lines. A more detailed phylogenetic relationship of the host insects representing local populations (Fig. S4) and that of the symbiotic bacteria representing local populations (Fig. S5) are available in the supplemental material. The significance of the phylogenetic congruence between the host insects and the symbiotic bacteria are indicated by different dotted lines.

We then collected diverse cassidine beetles representing 6 genera and 24 species in Japan (see Table S1 in the supplemental material), which were subjected to PCR amplification and sequencing of 16S rRNA, *gyrB*, and *groEL* genes of *Stammera*. For the majority of the samples, the symbiont genes were detected (Table S1). Molecular phylogenetic analyses supported the monophyly of *Stammera* symbionts of the diverse cassidine beetles with extremely AT-biased nucleotide compositions (Fig. S1 [Supplementary-material figS2]to S3). Comparison of the symbiont phylogeny with the host phylogeny revealed almost perfect congruence in the tree topologies, except for the placement of Laccoptera nepalensis (Fig. 3; Fig. S4 and S5). These results strongly suggested that (i) *Stammera* was acquired by the common ancestor of extant cassidine leaf beetles, (ii) *Stammera* has been stably maintained in the evolutionary course of cassidine leaf beetles via strict vertical transmission, (iii) the stability of *Stammera* has probably been underpinned by its important role of helping pectin digestion for the host beetles, and (iv) during the long symbiotic association over evolutionary time, the *Stammera* genome has been drastically reduced and specialized for the specific biological function, as highlighted in previous studies (22, 47).

10.1128/mbio.03691-21.1 FIG S1 Phylogenetic relationship of *Stammera* symbionts of cassidine leaf beetles based on bacterial 16S rRNA gene sequences. A maximum-likelihood phylogeny inferred from 1,605 aligned nucleotide sites is shown. The bootstrap probability value of maximum-likelihood analysis and the posterior probability value of Bayesian analysis are indicated at each node. Sequence accession numbers are in brackets, followed by adenine-thymine content percentages. Download FIG S1, PDF file, 1.2 MB. Copyright © 2022 Fukumori et al. 2022 Fukumori et al. https://creativecommons.org/licenses/by/4.0/ This content is distributed under the terms of the Creative Commons Attribution 4.0 International license.

10.1128/mbio.03691-21.2 FIG S2 Phylogenetic relationship of *Stammera* symbionts of cassidine leaf beetles based on bacterial *gyrB* gene sequences. A maximum-likelihood phylogeny inferred from 903 aligned nucleotide sites is shown. The bootstrap probability value of maximum-likelihood analysis and the posterior probability value of Bayesian analysis are indicated at each node. Sequence accession numbers are in brackets, followed by adenine-thymine content percentages. Download FIG S2, PDF file, 1.2 MB. Copyright © 2022 Fukumori et al. 2022 Fukumori et al. https://creativecommons.org/licenses/by/4.0/ This content is distributed under the terms of the Creative Commons Attribution 4.0 International license.

10.1128/mbio.03691-21.3 FIG S3 Phylogenetic relationship of *Stammera* symbionts of cassidine leaf beetles based on bacterial *groEL* gene sequences. A maximum-likelihood phylogeny inferred from 1,443 aligned nucleotide sites is shown. The bootstrap probability value of maximum-likelihood analysis and the posterior probability value of Bayesian analysis are indicated at each node. Sequence accession numbers are in brackets, followed by adenine-thymine content percentages. Note that *groEL* of *C. murraea* is treated as missing data. Download FIG S3, PDF file, 1.0 MB. Copyright © 2022 Fukumori et al. 2022 Fukumori et al. https://creativecommons.org/licenses/by/4.0/ This content is distributed under the terms of the Creative Commons Attribution 4.0 International license.

10.1128/mbio.03691-21.4 FIG S4 Phylogenetic relationship of cassidine leaf beetles based on concatenated sequences of the mitochondrial cytochrome oxidase I gene, the mitochondrial 16S rRNA gene, and the nuclear 28S rRNA gene. A maximum-likelihood phylogeny inferred from 785, 550, and 804 aligned nucleotide sites of the cytochrome oxidase I gene, the mitochondrial 16S rRNA gene, and the nuclear 28S rRNA gene, respectively, is shown. The bootstrap probability value of maximum-likelihood analysis and the posterior probability value of Bayesian analysis are indicated at each node. Sequence accession numbers are in brackets. Download FIG S4, PDF file, 0.9 MB. Copyright © 2022 Fukumori et al. 2022 Fukumori et al. https://creativecommons.org/licenses/by/4.0/ This content is distributed under the terms of the Creative Commons Attribution 4.0 International license.

10.1128/mbio.03691-21.5 FIG S5 Phylogenetic relationship of *Stammera* symbionts of cassidine leaf beetles based on concatenated sequences of the bacterial 16S rRNA gene, the *gyrB* gene, and the *groEL* gene. A maximum-likelihood phylogeny inferred from 1,549, 903, and 1,443 aligned nucleotide sites of the bacterial 16S rRNA gene, the *gyrB* gene, and the *groEL* gene, respectively, is shown. The bootstrap probability value of maximum-likelihood analysis and the posterior probability value of Bayesian analysis are indicated at each node. Sequence accession numbers are in brackets. Note that *groEL* of *C. murraea* is treated as missing data. Download FIG S5, PDF file, 0.6 MB. Copyright © 2022 Fukumori et al. 2022 Fukumori et al. https://creativecommons.org/licenses/by/4.0/ This content is distributed under the terms of the Creative Commons Attribution 4.0 International license.

10.1128/mbio.03691-21.7 TABLE S1 Samples of cassidine leaf beetles examined in this study, their symbiotic organs, and sequence accession numbers of host and symbiont genes. Download Table S1, XLSX file, 0.02 MB. Copyright © 2022 Fukumori et al. 2022 Fukumori et al. https://creativecommons.org/licenses/by/4.0/ This content is distributed under the terms of the Creative Commons Attribution 4.0 International license.

### Repeated symbiont losses despite the conserved cassidine-*Stammera* association.

However, we found that *Stammera* was not detected in 3 of the 24 cassidine species we examined, namely, *C. nebulosa*, Cassida obtusata, and Thlaspida lewisii (Table S1). Mapped on the host phylogeny, it was estimated that the *Stammera* infection must have been lost three times independently (Fig. 3). Considering the important digestive role of the symbiont and the long-lasting host-symbiont association (22, 47), the recurrent losses of *Stammera* were unexpected as well as striking. Meanwhile, it should be noted that in his pioneer report, Stammer (16) already described that neither symbiotic bacteria nor symbiotic organs were found in 2 of 7 cassidine species he examined, *C. nebulosa* and *C. flaveola*.

### Histological inspection of symbiotic organs and *Stammera* infection among cassidine leaf beetles.

**FIG 4:**
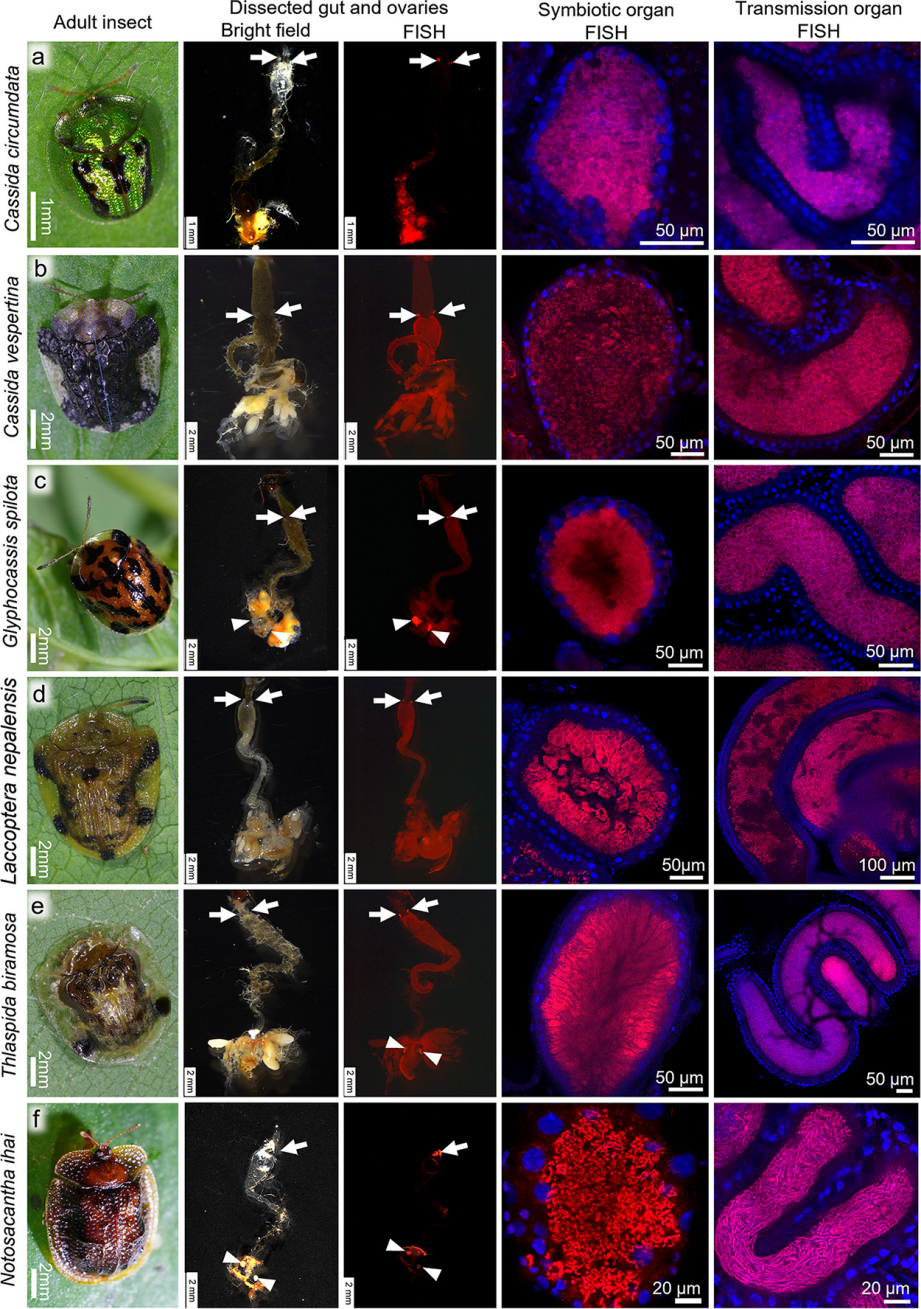
Symbiotic system of diverse cassidine leaf beetles. (a) *Cassida circumdata*. (b) *Cassida vespertina*. (c) *Glyphocassis spilota*. (d) *Laccoptera nepalensis*. (e) *Thlaspida biramosa*. (f) *Notosacantha ihai*. Panels from left to right: adult insects, bright-field images of dissected gut and ovaries, whole-mount FISH images of dissected gut and ovaries, confocal FISH images of gut-associated symbiotic organ, and confocal FISH images of ovary-associated transmission organ, respectively. Arrows and arrowheads indicate gut-associated symbiotic organs and ovary-associated transmission organs, respectively. In FISH images, red signals show bacterial 16S rRNA, whereas blue signals show host nuclear DNA. For FISH, the general bacterial probe Al555-EUB338 (third row) and the *Stammera*-specific probe Al555-Tor971 (fourth and fifth rows) were used. See also Fig. S6 in the supplemental material.

**FIG 5:**
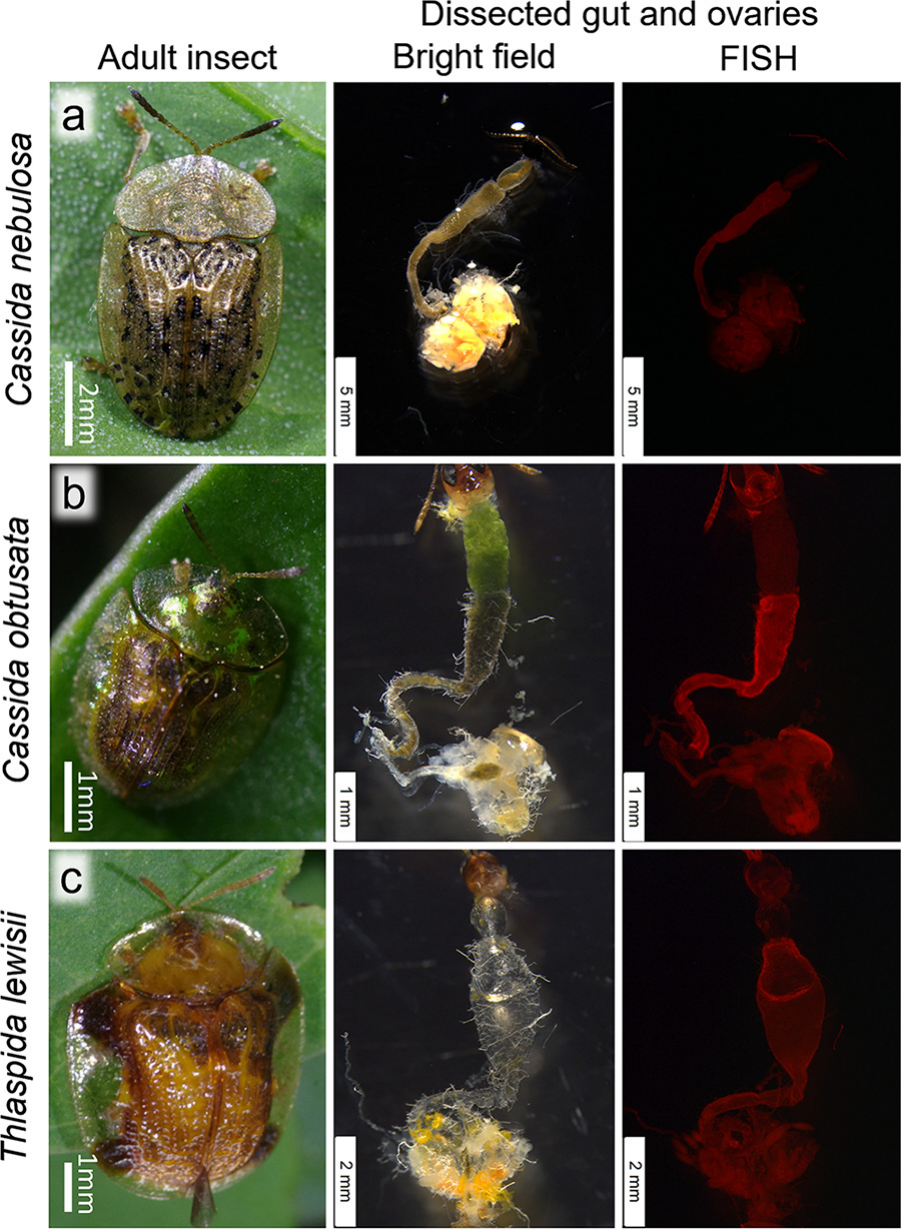
Cassidine leaf beetles in which neither *Stammera* infection nor symbiotic organs were detected. (a) *Cassida nebulosa*. (b) *Cassida obtusata*. (c) *Thiaspida lewisii*. Panels from left to right: adult insects, bright-field images of dissected gut and ovaries, and whole-mount FISH images of dissected gut and ovaries, respectively. For FISH, the general bacterial probe Al555-EUB338 was used.

Hence, we histologically inspected the diverse cassidine species for the presence of the symbiotic organs and the localization of the symbiotic bacteria. In all the *Stammera*-positive species (2 *Aspidimorpha* species, 14 *Cassida* species, Glyphicassis spilota, Laccoptera quadrimaculata, 2 *Notosacantha* species, and Thlaspida biramosa), FISH identified the symbiont localization in the symbiotic organs at the foregut-midgut junction and also in the genital accessory organ in reproductively mature female specimens (Fig. 4; Fig. S6). In the *Stammera*-negative species (*C. nebulosa*, *C. obtusata*, and *T. lewisii*), in contrast, neither the symbiotic organs nor the FISH signals of the symbiont were detected (Fig. 5). These observations verified the PCR, sequencing, and phylogenetic results regarding the absence of *Stammera* in *C. nebulosa*, *C. obtusata*, and *T. lewisii* (Fig. 3; Table S1) and also confirmed the early report that neither symbiotic bacteria nor symbiotic organs were found in *C. nebulosa* (16).

10.1128/mbio.03691-21.6 FIG S6 Symbiotic system of diverse cassidine leaf beetles (continued). (a) *Cassida versicolor*. (b) *Cassida murraea*. (c) *Cassida lineola*. (d) *Cassida mongolica*. (e) *Cassida piperata*. (f) *Notosacantha nishiyamai*. Panels from left to right: adult insects, bright-field images of dissected gut and ovaries, whole-mount FISH images of dissected gut and ovaries, confocal FISH images of gut-associated symbiotic organ, and confocal FISH images of ovary-associated transmission organ, respectively. Arrows and arrowheads indicate gut-associated symbiotic organs and ovary-associated transmission organs, respectively. In FISH images, red signals show bacterial 16S rRNA, whereas blue signals show host nuclear DNA. For FISH, the general bacterial probe Al555-EUB338 (third row) and the *Stammera*-specific probe Al555-Tor971 (fourth and fifth rows) were used. See also Fig. 4. Download FIG S6, PDF file, 11.2 MB. Copyright © 2022 Fukumori et al. 2022 Fukumori et al. https://creativecommons.org/licenses/by/4.0/ This content is distributed under the terms of the Creative Commons Attribution 4.0 International license.

### Two or four: diversity in symbiotic organs of cassidine leaf beetles.

**FIG 6:**
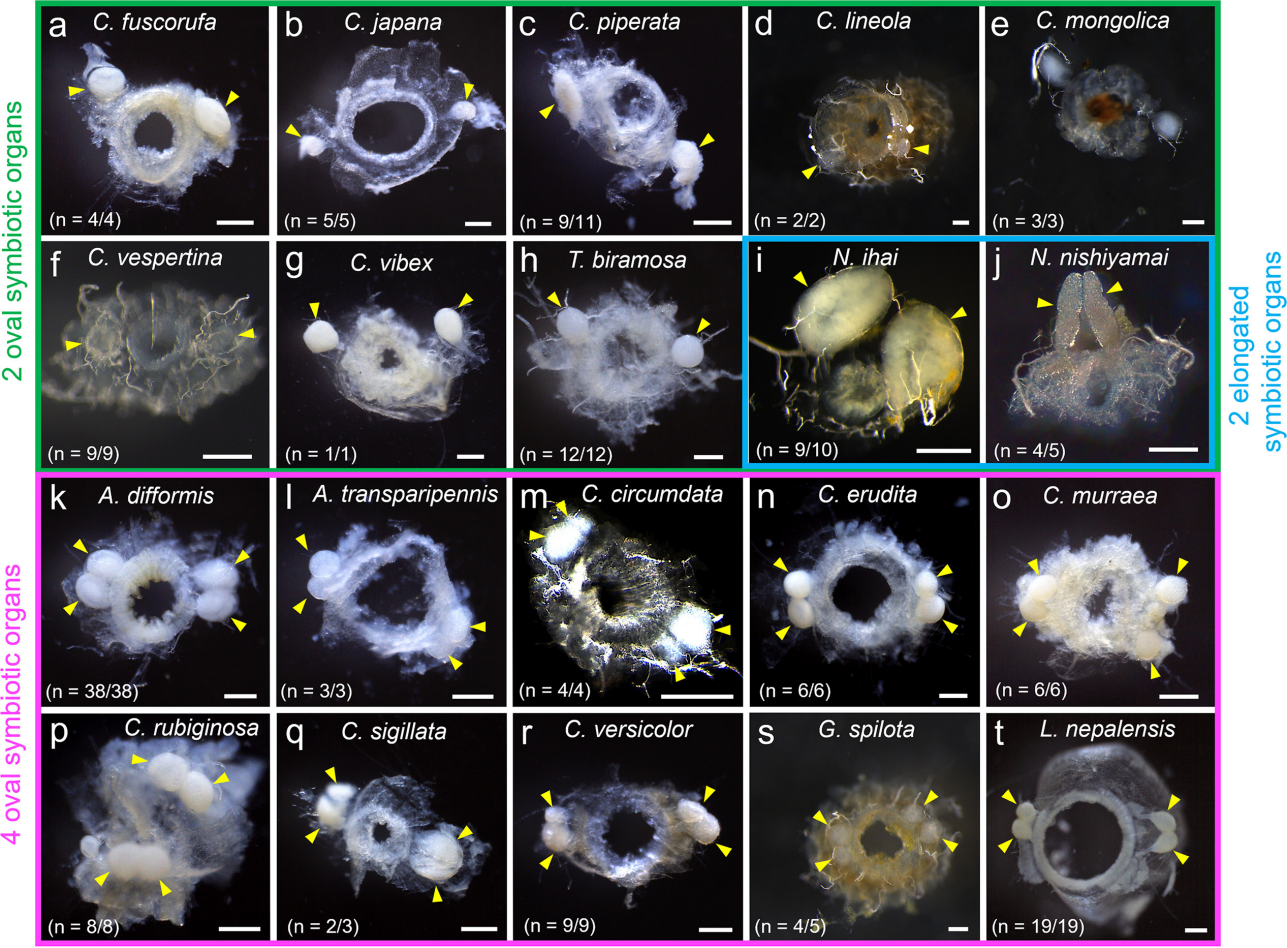
Symbiotic organs on the dissected foregut-midgut junction of cassidine leaf beetles. (a to j) Species with two symbiotic organs, highlighted by a green outline. (a) *Cassida fuscorufa*. (b) *Cassida japana*. (c) *Cassida piperata*. (d) *Cassida lineola*. (e) *Cassida mongolica*. (f) *Cassida vespertina*. (g) *Cassida vibex*. (h) *Thiaspida biramosa*. (i) *Notosacantha ihai*. (j) *Notosacantha nishiyamai*. For panels i and j, species with larger and elongated symbiotic organs are further highlighted by a blue outline. (k to t) Species with four symbiotic organs, highlighted by a magenta outline. (k) *Aspidimorpha difformis*. (l) *Aspidimorpha transparipennis*. (m) *Cassida circumdata*. (n) *Cassida erudita*. (o) *Cassida murraea*. (p) *Cassida rubigonosa*. (q) *Cassida sigillata*. (r) *Cassida versicolor*. (s) *Glyphocassis spilota*. (t) *Laccoptera nepalensis*. Each arrowhead indicates a symbiotic organ. Bars, 200 μm. As for sample size, for example, *n* = 2/3 means that of 3 individuals inspected, 2 individuals exhibited the symbiotic organs. It should be noted that in some of the samples, the symbiotic organs were either degenerate or difficult to recognize due to poor morphological preservation of fixed/frozen samples. For more details, see Table S1.

We found that, notably, the symbiotic organs of the cassidine leaf beetles exhibit remarkable diversity in number, shape, and structure. Cassida fuscorufa, Cassida japana, Cassida piperata, Cassida lineola, Cassida mongorica, Cassida vespertina, Cassida vibex, and *T. biramosa* possessed two symbiotic organs, with an oval symbiotic organ being located on each side of the foregut-midgut junction (Fig. 6a to h). Notosacantha ihai and Notosacantha nishiyamai also had two symbiotic organs at the foregut-midgut junction, although each symbiotic organ was larger in size and elongated in shape (Fig. 6i and j). On the other hand, *A. difformis*, Aspidimorpha transparipennis, Cassida circumdata, Cassida erudita, Cassida murraea, *C. rubiginosa*, Cassida sigillata, Cassida versicolor, *G. spilota*, and *L. nepalensis* developed four symbiotic organs, with two oval symbiotic organs being located on each side of the foregut-midgut junction (Fig. 6k to t). Considering that the congenic *Cassida* species possess either two or four symbiotic organs, it seemed that the number of symbiotic organs may have experienced complex evolutionary trajectories in the cassidine leaf beetles.

### Discovery of vestigial symbiotic organs in *C. nebulosa*, *C. obtusata*, and *T. lewisii*.

**FIG 7:**
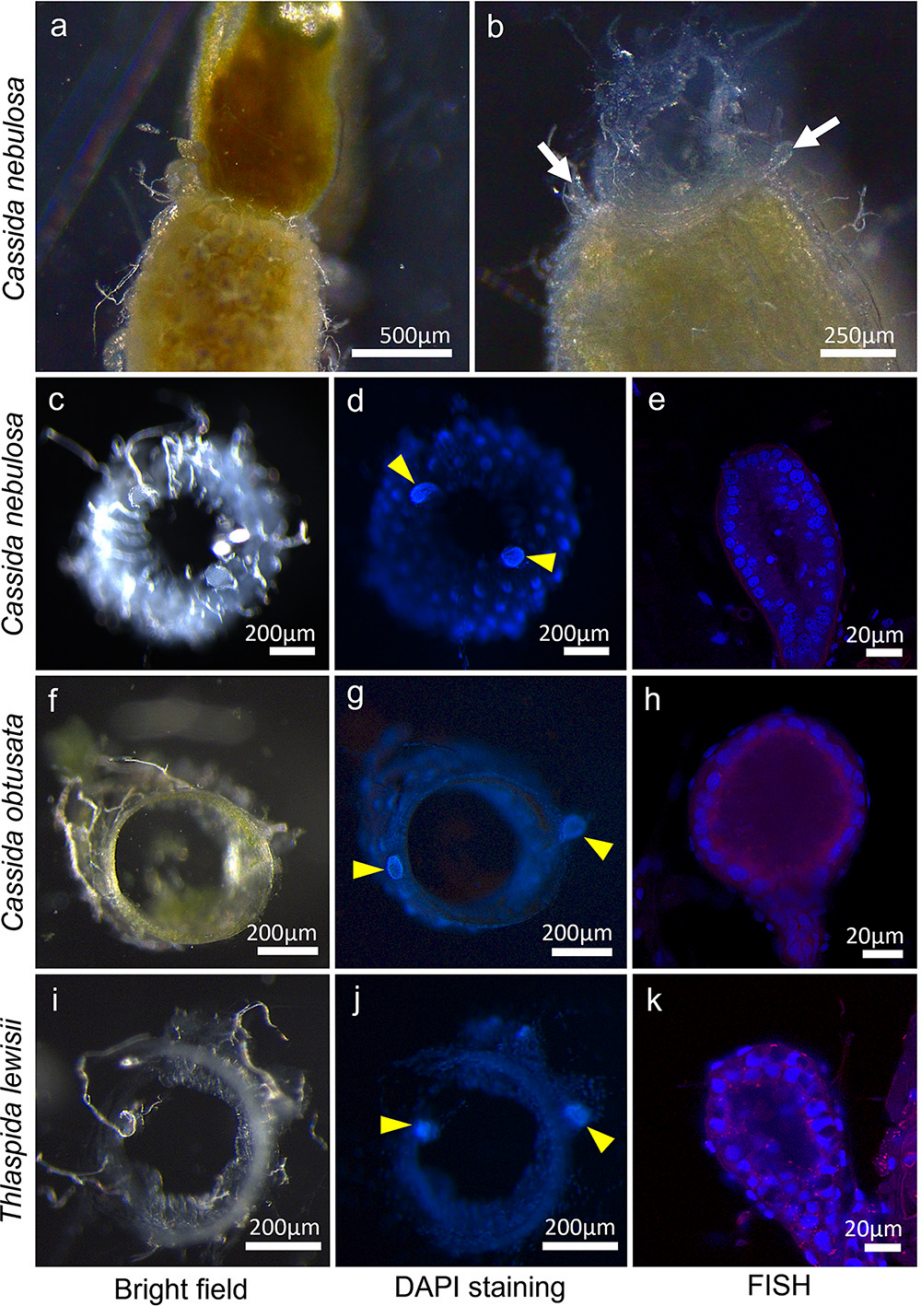
Vestigial symbiotic organs identified in cassidine leaf beetles devoid of *Stammera* infection. (a to e) *Cassida nebulosa*. (f to h) *Cassida obtusata*. (i to k) *Thlaspida lewisii*. (a) Side view of a dissected foregut-midgut junction. No symbiotic organs are seen. (b) Side view of the dissected foregut-midgut junction, from which the foregut is pulled and removed with forceps. Vestigial symbiotic organs are exposed on both sides of the junction. (c, f, and i) Bright-field images of the cross-sectioned foregut-midgut junction in which the vestigial symbiotic organs are difficult to recognize. (d, g, and j) Fluorescence images of the same specimens shown in panels c, f, and i in which the vestigial symbiotic organs are clearly visualized by DAPI staining based on small and dense nuclear DNA signals. (e, h, and k) Confocal FISH images of the vestigial symbiotic organs whose inner cavities exhibit no dense bacterial signals. For FISH, the general bacterial probe Al555-EUB338 was used.

Our initial histological observations (Fig. 5) in combination with the previous report (16) indicated that *C. nebulosa* lacks not only the *Stammera* infection but also the gut-associated symbiotic organs. However, our close inspection of the dissected alimentary tract uncovered that *C. nebulosa* actually retains a pair of vestigial symbiotic organs at the foregut-midgut junction. Though hard to recognize in the dissected alimentary tract (Fig. 7a), when the foregut was either carefully pulled up or removed, tiny paired projections were exposed (Fig. 7b). Though not readily recognizable in the freshly excised foregut-midgut junction (Fig. 7c), DNA staining highlighted a dense assemblage of small nuclei of the paired structures that were distinct from larger nuclei of the gut epithelial cells (Fig. 7d). FISH observations revealed that each of the tiny projections was a pouch-like structure consisting of an epithelial cell layer just like the symbiotic organ, although its inner cavity was devoid of the *Stammera* infection (Fig. 7e). Similar structures were also identified in *C. obtusata* and *T. lewisii* (Fig. 7f to k). No major symbiotic bacteria in place of *Stammera* were detected in *C. nebulosa*, *C. obtusata*, or *T. lewisii*, excluding the possibility of symbiont replacement in these cassidine lineages. These observations uncovered that the symbiotic organs have been developmentally and evolutionarily maintained even after the symbiont losses in the cassidine leaf beetles.

### Evolutionary dynamics of symbiotic organs in cassidine leaf beetles.

**FIG 8:**
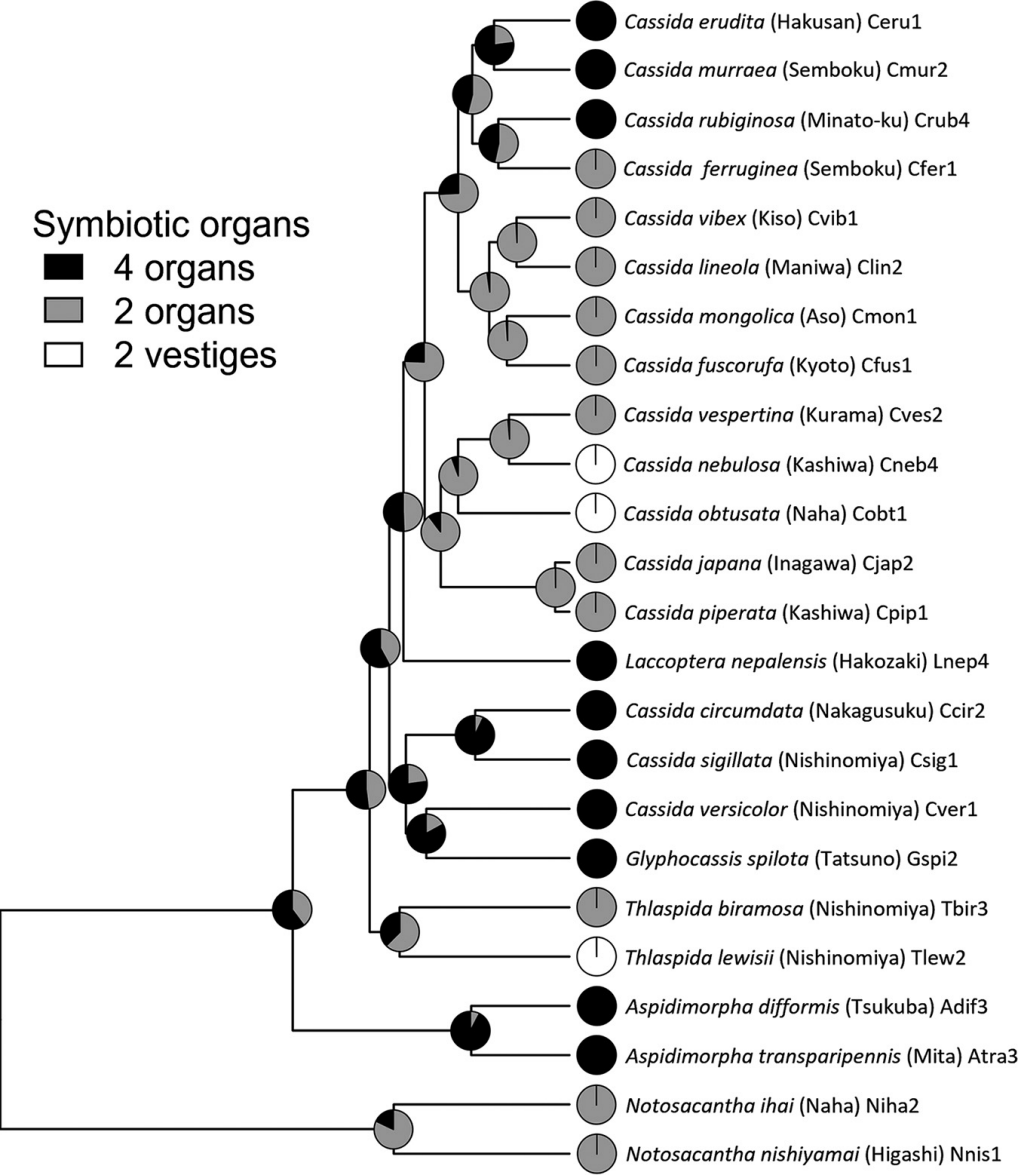
Maximum-likelihood reconstruction of the number of symbiotic organs and losses of *Stammera* infection in the evolutionary course of cassidine leaf beetles using Mesquite version 1.1 (93). Each pie graph indicates the maximum-likelihood support for the ancestral state at each node.

On the basis of these results, we mapped the numbers of symbiotic organs and symbiont losses on the host phylogeny and estimated the evolutionary process of the symbiotic organs in the cassidine leaf beetles. Both Bayesian and maximum parsimony analyses highlighted the dynamic evolution of the symbiotic organs that have repeatedly switched between two and four in number (Fig. 8). The evolutionary trajectory was so complex that it was not feasible to reliably infer the ancestral number of the symbiotic organs either as two or four in the common ancestor of the cassidine leaf beetles we examined (Fig. 8).

## DISCUSSION

In this study, we investigated the symbiotic bacteria and symbiotic organs of 24 Japanese cassidine leaf beetles in detail, which uncovered repeated symbiont losses and recurrent structural switching of the symbiotic organs between two and four in number (Fig. 3 and 8). In a recent microbial genomic study, the world’s cassidine leaf beetles, representing 13 species and 10 genera, were subjected to *Stammera* genome sequencing, which revealed extremely reduced symbiont genomes of 0.3 Mb or smaller in size, conserved symbiont functioning specialized for production of pectinases, and conserved host-symbiont association and cospeciation over evolutionary time (47). In this context, our finding of the recurrent evolutionary losses of *Stammera* is striking. Why and how are these cassidine species, *C. nebulosa*, *C. obtusata*, and *T. lewisii*, able to survive in the absence of the highly conserved and seemingly essential digestive symbiont *Stammera*? The following four hypotheses are conceivable to account for the symbiont losses: (i) host plant shift, (ii) symbiont replacement, (iii) horizontal gene acquisition of pectinases from the symbiont or other microbial sources, and (iv) *de novo* evolution of pectinases via cooption and neo- or subfunctionalization of preexisting genes for degradation of plant polysaccharides. The first hypothesis assumes that the host’s utilization of a new food plant containing less pectin may have facilitated the symbiont loss. However, this scenario is unlikely at least for the symbiont loss in *C. nebulosa*, on the grounds that *Stammera*-harboring *C. piperata* and *Stammera*-free *C. nebulosa* commonly feed, grow, and reproduce on the same plant, Chenopodium album (see Table S1 in the supplemental material). The second hypothesis is also unlikely because no other microbial symbionts were detected in *C. nebulosa*, *C. obtusata*, and *T. lewisii* (Fig. 7e, h, and k), although possible involvement of minor microbial associates cannot be excluded. Hence, we suspect that either the third or fourth hypothesis may account for the *Stammera* losses in these cassidine lineages. Future studies should be directed to genomic, transcriptomic, and proteomic surveys of prokaryotic and eukaryotic pectinases and other cell wall-degrading enzymes in these and other cassidine species. It should be noted that genomic and transcriptomic surveys of the major herbivorous insect groups Chrysomeloidea (leaf beetles, longicorn beetles, and allies) and Curculionoidea (weevils and allies) revealed recurrent acquisitions of genes for decomposing cellulose, hemicellulose, and pectin via lateral gene transfers from fungi and bacteria (48–50).

We demonstrated that the number of symbiotic organs has changed between two and four in a dynamic manner during the evolutionary course of cassidine leaf beetles. Due to complex evolutionary patterns, the ancestral state of the symbiotic organs, either two or four, cannot be estimated reliably (Fig. 8). In diverse insects, multiple blind sac-like structures, called gastric ceca, are present at the foregut-midgut junction, and their biological functions are inferred to increase the inner surface area of the alimentary tract for facilitating digestion and absorption (51). In the genetic model insect Drosophila melanogaster, larvae possess four gastric ceca, whose morphogenesis has been investigated using developmental and molecular genetic approaches (52–55). In several insect groups, including leaf beetles, four gastric ceca have been reported to function as symbiotic organs: four voluminous gastric ceca harboring the symbiotic bacteria *Macropleicola* spp. in larvae of Donaciinae leaf beetles (15, 23), four round gastric ceca hosting symbiotic bacteria in larvae of a *Bromius* leaf beetle (16, 21), four voluminous gastric ceca populated by the symbiotic fungi *Symbiotaphrina* spp. in larvae of Anobiinae drugstore beetles (14, 17), and four round gastric ceca containing the symbiotic bacterium Erwinia dacicola in larvae of the olive fly Bactocera oleae (18, 56). On the other hand, it has been reported that the number of symbiotic gastric ceca is often various and/or changeable, as follows: the number and morphology of larval symbiotic gastric ceca exhibit remarkable variation among Cerambycidae longicorn beetles (57); toward pupation and metamorphosis of the Donaciinae leaf beetles, four larval symbiotic gastric ceca disappear and two of six Malpighian tubules are transformed into the symbiotic sites (15, 23); during metamorphosis of the *Bromius* leaf beetle, four round symbiotic gastric ceca in larvae are replaced by a number of finger-like symbiotic gastric ceca in adults (16, 21). In the light of these observations, it is conceivable, although speculative, that four gastric ceca might be the ancestral state of the symbiotic organs in the cassidine leaf beetles. However, more data on the diversity, the developmental processes, and the morphogenetic mechanisms of the symbiotic organs in cassidine leaf beetles should be compiled to obtain more reliable estimates. Functional and adaptive relevance of the interspecific differences in size and number of the symbiotic organs in the cassidine leaf beetles is currently elusive and deserves future studies.

In holometabolous insects, during metamorphosis from larva through pupa to adult, their tissues and organs are drastically reconstructed and reorganized (58), and the symbiotic organs are no exception. In weevils, for example, the well-developed symbiotic organ, the bacteriome, associated with the larval midgut disintegrates during metamorphosis and disappears in adults (59). Since adult weevils develop sclerotized cuticle for which much tyrosine is required, the symbiont is specialized for tyrosine provisioning (31). Hence, while the symbiotic organ is highly developed and metabolically active in larvae to accumulate sufficient tyrosine for adult cuticle formation, the symbiont functioning is no longer necessary upon adult emergence and onward, and thus the symbiotic organ is lost (60). In cassidine leaf beetles, in contrast, the symbiotic organs are retained not only in larvae but also in adults (Fig. 1), probably because they feed on the same host plant leaves, for which the symbionts are constantly needed for food digestion throughout the life stages.

The biological significance of the different number of the symbiotic organs, two or four, is currently elusive. The molecular mechanisms underlying the different number of the symbiotic organs are also elusive and to be pursued in future studies. In previous studies on aphid, seed bug, and ant, such homeodomain transcription factors as *Ultrabithorax*, *abdominala-A*, and *engrailed* have been demonstrated to be coopted for and involved in the formation of the symbiotic organs (61–63). In D. melanogaster, such transcription factors and morphogens as *Sex combs reduced*, *Ultrabithorax*, *Antennapedia*, *labial*, *decapentaplegic*, and *wingless*, and also matrix metalloproteinases and autophagy, have been shown to be involved in the development of four larval gastric ceca (52–55). Considering that transitions between two and four symbiotic organs in cassidine leaf beetles can be viewed as dorsoventral duplication or fusion of the symbiotic organs, although speculative, the involvement of dorsoventral patterning genes such as Toll pathway genes, *dorsal*, *decapentaplegic*, *short gastrulation*, and others seems plausible (64, 65). Transcriptomics and RNA interference of these genes in the developmental course of the symbiotic organs of cassidine leaf beetles are to be conducted in future studies.

Despite the evolutionary symbiont losses, the paired symbiotic organs were retained, although atrophied, in the distinct cassidine lineages *C. nebulosa*, *C. obtusata*, and *T. lewisii* (Fig. 3, 5, 7, and 8). These observations suggest that (i) the developmental program of the symbiotic organs must be ancient, plausibly originating from the common ancestor of the extant cassidine species, (ii) the symbiont losses in *C. nebulosa*, *C. obtusata*, and *T. lewisii* must have occurred recently in the respective lineages independently, and (iii) therefore, the symbiotic organs are formed even in the absence of the symbiotic bacteria due to, as it were, developmental inertia. A similar phenomenon was experimentally shown in the saw-toothed grain beetle Oryzaephilus surinamensis, with abdominal bacteriomes harboring “*Candidatus* Shikimatogenerans silvanidophilus” (29, 66). By high-temperature treatment and subsequent rearing on nutritionally rich whole wheat flour, a symbiont-free strain of *O. surinamensis* was established and maintained, and it continued to form atrophied sterile bacteriomes for 25 generations (67, 68). In previous studies, histological observations of diverse insect-microbe symbiotic associations revealed that symbiont degeneration and/or loss tends to occur in a male-specific manner in aphids (61, 69–71), scale insects (72, 73), lice (74, 75), leaf beetles (15, 23), powderpost beetles (30, 76), and others (18), some of which also entail degeneration and/or loss of the symbiotic organs. Adult male insects do not grow, produce no eggs, and do not transmit the symbionts to the next generation, which plausibly account for the recurrent evolution of the male-specific symbiont losses. Whether and how the symbiont losses are relevant to the degeneration and loss of the symbiotic organs is elusive in most cases. Notably, however, using bacteriocyte-specific transcription factor genes as molecular markers, Braendle et al. (61) reported interesting observations on several gall-forming social aphids. In Tuberaphis styraci (Aphidoidea: Hormaphididae: Cerataphidini) that has lost *Buchnera* and bacteriocytes and harbors a fungal symbiont in the body cavity extracellularly (39, 40), the cells destined to be bacteriocytes are transiently formed during embryogenesis (61). In contrast, in Pemphigus spyrothecae (Aphidoidea: Pemphigidae: Pemphigini) that has lost *Buchnera* and bacteriocytes in a male-specific manner (70, 71), no cells to be bacteriocytes appeared during male embryogenesis (61). The difference between *T. styraci* and *P. spyrothecae* may be attributable to the ancientness of their symbiont losses: the symbiont replacement of *Buchnera* by the fungal symbiont must have occurred within the aphid tribe Cerataphidini (18, 39), whereas the male-specific symbiont absence may be commonly observed across the aphid family Pemphigidae (18, 70, 71).

Here we provide several phylogenetic and systematic notes. Our data showed that the clade of *Cassida* spp. contains *G. spilota* and *L. nepalensis* (Fig. 3; Fig. S4). There are two possible explanations for these phylogenetic patterns. One possibility is that although morphologically deviated, *G. spilosa* and *L. neparensis* are placed within the *Cassida* clade and their taxonomic treatment should be reconsidered. Another possibility is that some evolutionary events like mitochondrial introgression have caused the phylogenetic anomaly. Our data also showed that the symbiont phylogeny is highly congruent with the host phylogeny, except for the placement of *L. nepalensis* (Fig. 3). This exception may be accounted for either by lateral transfer of the symbiont across the host lineages or by mitochondrial introgression of the host side. Future phylogenetic analyses with more sequence data are anticipated to address these systematic uncertainties.

In conclusion, our findings highlight dynamic evolutionary aspects of mutualistic insect-microbe associations in cassidine leaf beetles, which provide a promising model system to investigate how such symbiotic systems have been established, diversified, and degenerated in the course of host-symbiont coevolution. Considering that the Cassidinae *sensu stricto* consists of about 3,000 species, 150 genera, and 20 tribes in the world (46), the evolutionary events we identified here must be only the tip of an iceberg. We expect that a wider survey of the world’s diversity of cassidine leaf beetles will uncover much more dynamic aspects of the evolution of symbiosis.

## MATERIALS AND METHODS

### Insect materials.

The samples of cassidine leaf beetles are described in Table S1 in the supplemental material. The insects were immediately used for experiments or preserved in 99.5% ethanol, in acetone, or in an ultracold freezer at −80°C until use. The insects were dissected in 70% ethanol or phosphate-buffered saline (PBS; 0.8% NaCl, 0.02% KCl, 0.115% Na_2_HPO_4_, 0.02% KH_2_PO_4_, pH 7.4) by using fine tweezers under a dissection microscope (M165FC; Leica).

### FISH.

Whole-mount fluorescent *in situ* hybridization (FISH) targeting bacterial 16S rRNA was performed essentially as described previously (77). The dissected insect tissues were fixed in Carnoy’s solution (60% ethanol, 30% chloroform, 10% acetic acid) for 15 min, washed three times in 70% ethanol, and subsequently washed three times in PBSTx (0.3% Triton X-100 in PBS). Alternatively, the dissected insect tissues were fixed in 4% paraformaldehyde solution in PBS for 3 h and then thoroughly washed in PBS. The fixed insect tissues were washed twice in a hybridization buffer (20 mM Tris-HCl [pH 8.0], 0.9 M NaCl, 0.01% SDS, 30% formamide). To specifically target 16S rRNA of the *Stammera* symbiont of cassidine leaf beetles, the oligonucleotide probe Tor971 (5′- CCA GGT AAG GTT CTT CGC GT-3′) was labeled with fluorochrome Alexa Fluor 555 (Thermo Fisher Scientific, USA) at the 5′ terminus. For universal detection of bacterial 16S rRNA, the probe EUB338 (5′-GCT GCC TCC CGT AGG AGT-3′) (78) 5′-end labeled with Alexa Fluor 555 was also used. The tissue samples were incubated in hybridization buffer containing a 50 nM concentration of the probe and 4.5 μM 4′,6-diamidino-2-phenylindole (DAPI) (Thermo Fisher Scientific) overnight at room temperature, washed thoroughly in PBSTx, mounted in SlowFade gold antifade solution (Thermo Fisher Scientific), and observed under an epifluorescence microscope (Axiophot; Carl Zeiss, Germany) and a laser scanning confocal microscope (LSM 700; Carl Zeiss, Germany). For FISH control experiments, (i) no probe controls, (ii) competition controls in which unlabeled oligonucleotides were added to the hybridization buffer to suppress the fluorescent signals, and (iii) RNase digestion controls in which the tissue samples were treated with RNase A prior to hybridization were conducted.

### Transmission electron microscopy.

The gut-associated symbiotic organs and the female-specific genital accessory organs were dissected in 0.1 M phosphate buffer (pH 7.4) containing 2.5% glutaraldehyde, prefixed in the fixative at 4°C for 5 h, and postfixed in 2% osmium tetroxide at 4°C for 90 min. After dehydration through an ethanol series, the tissues were embedded in Epon812 resin and cut into ultrathin sections (thickness, 70 nm) using an ultramicrotome (EM UC7; Leica, Germany). The sections were stained with uranyl acetate and lead citrate and observed under a transmission electron microscope (model H-7600; Hitachi, Japan).

### DNA analysis.

The dissected tissues were individually subjected to DNA extraction using a QIAamp DNA mini kit or a QIAamp DNA micro kit (Qiagen, Netherlands). Bacterial genes were amplified by PCR using Ex Taq DNA polymerase (TaKaRa Bio, Japan) or Gflex DNA polymerase (TaKaRa Bio) with the primers 16SA1 (5′-AGA GTT TGA TCM TGG CTC AG-3′) (79) and 1507R (5′-TAC CTT GTT ACG ACT TCA CCC CAG-3′) (80) for the 16S rRNA gene, gyrBsymF (5′-TTA TCA TGA CWG TAT TAC ATG CWG G-3′) (81) and gyrBsymR (5′-TCC AGC WGA ATC WCC TTC WAC-3′) (81) for the *gyrB* gene, and groEL_Tor1F (5′-ATG GCA GCT AAA GAT GTA AAG TTT-3′) and groEL_Tor1R (5′-AAC CTG CAA CAG ATG AAG CA-3′) for the *groEL* gene. After successful amplification was checked by electrophoresis on 1% agarose gels, each PCR product was purified using exonuclease I (New England Biolabs, USA) and shrimp alkaline phosphatase (TaKaRa Bio) at 37°C for 15 min, followed by 80°C for 15 min. The purified PCR products were directly subjected to a sequencing reaction using the BigDye terminator v3.1 cycle sequencing kit (Thermo Fisher Scientific) and analyzed by a 3130xl genetic analyzer (Thermo Fisher Scientific). The internal primer 16SA2 (5′-GTG CCA GCA GCC GCG GTA ATA C-3′) (80) was used for sequencing of the bacterial 16S rRNA gene. For sequencing of the bacterial *groEL* gene, the internal specific primers groEL_Tor4F (5′-AGT TGC TGC TGG TAT GAA TCC T-3′), groEL_Tor5F (5′-GCT GAA GAT GTT GAA GGA GAA GC-3′), and groEL_Tor3R (5′-CCA GGA GCT TTA ACT GCA GC-3′) were also used.

In addition, host genes were similarly amplified with the primers COS2183N (5′ -CAR CAY YTA TTY TGR TTY TTY GG-3′) and COA3107 (5′-TCT ATT ARD GGD GAD GCD CTA TCT TG-3′) (82) for the mitochondrial cytochrome oxidase subunit I (COI) gene, 16sar (5′-CGC CTG TTT ATC AAA AAC AT-3′) and 16Sbr (5′-CCG GTC TGA ACT CAG ATC ACG T-3′) (83) for the 16S rRNA gene, and 28S-01 (5′-GAC TAC CCC CTG AAT TTA AGC AT-3′) and 28SR-01 (5′-GAC TCC TTG GTC CGT GTT TCA AG-3′) (84) for the 28S gene. After checking successful amplification by electrophoresis on 1% agarose gels, we conducted the same procedure with bacterial genes.

### Molecular phylogenetic analyses.

Multiple alignments of the nucleotide sequences were generated using MAFFT v7.427 (85). The alignments were then inspected and corrected manually, and ambiguously aligned sites were removed. We conducted phylogenetic analyses based on maximum likelihood by using RAxML-NG (86) to construct species trees of symbionts and hosts for cocladogenesis analyses, where the sequence data were not partitioned by codon positions. The optimum substitution models for each of the symbiont data sets were estimated by ModelTest-NG (87, 88) on the basis of the Akaike information criterion. The selected models are listed in Table S2. Bootstraps were conducted with 1,000 iterations. We also conducted phylogenetic analyses based on a Bayesian method using BEAST v.1.10.4 (89). The optimum substitution models for each of the data sets were estimated by Kakusan4 (90) on the basis of the Bayesian information criterion. The selected models are listed in Table S2. As for symbionts, a proportional model among codons was selected for both phylogenetic analyses, including the regional populations, and the cocladogenesis analysis. We also constructed phylogenetic trees including regional populations of each species using each gene region separately with the optimum substitution models estimated using Kakusan4. A codon separate model was selected for *gyrB* and *groEL*. As for hosts, a proportional model among codons was selected for all phylogenetic analyses using BEAST. When a codon proportional model was selected by Kakusan4, we partitioned the data for protein coding regions (*gyrB*, *groEL*, and *COI*) by codon position by linking the parameters for substitution rate, rate heterogeneity, and base frequency among codon positions in the analysis. We used the random local clock model with the Yule process as the tree prior to the phylogenetic analyses used for the cocladogenesis analysis and used the log-normal relaxed clock model with the constant size as the tree prior to the phylogenetic analyses including regional populations of each species. Markov chain Monte Carlo chains were run for 100,000,000 generations, with trees sampled every 10,000 generations. We constructed a maximum clade credibility tree by using a burn-in of 25% with TreeAnnotator v.1.10.4 (89). We checked the convergence of parameters using Tracer 1.7.1 (91).

10.1128/mbio.03691-21.8 TABLE S2 Selected models for molecular phylogenetic analyses. Download Table S2, XLSX file, 0.01 MB. Copyright © 2022 Fukumori et al. 2022 Fukumori et al. https://creativecommons.org/licenses/by/4.0/ This content is distributed under the terms of the Creative Commons Attribution 4.0 International license.

### Ancestral state reconstruction.

We inferred the ancestral states of the symbiotic organs at ancestral nodes of the host beetles by the maximum likelihood method using Mesquite version 3.61 (92). We set three states for symbiotic organs (4 organs, 2 organs, or 2 vestiges). We used the phylogenetic tree for cocladogenesis analysis estimated by BEAST.

### Cospeciation analysis.

The levels of the host-symbiont phylogenetic congruence were evaluated by a distance-based method, ParaFit (93), implemented in CopyCat (94). ParaFit was used to test the null hypothesis of random association between host and symbiont data sets. Tests of random association were performed with 99,999 permutations globally across both matrices of the host-symbiont association.
