# Supplementary figures and images for: Evolutionary Dynamics of Host Organs for Microbial Symbiosis in Tortoise Leaf Beetles (Coleoptera: Chrysomelidae: Cassidinae)
Source: mBio. 2022 Jan 25;13(1):e03691-21. doi: 10.1128/mbio.03691-21 (PMC8787481; doi:10.1128/mbio.03691-21)

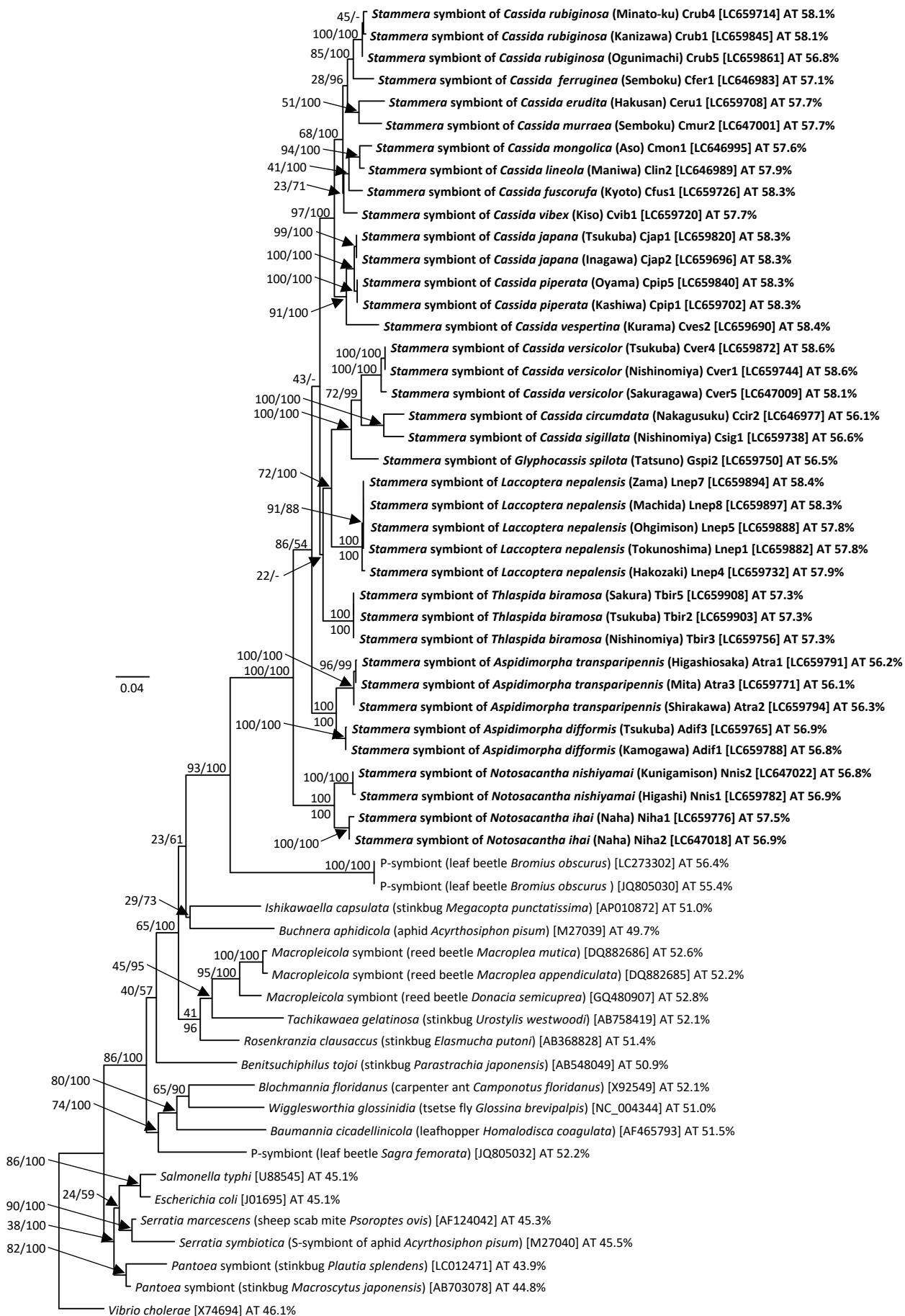

Fig. S1

Supplement: FIG S1 [file mbio.03691-21-sf001.pdf]

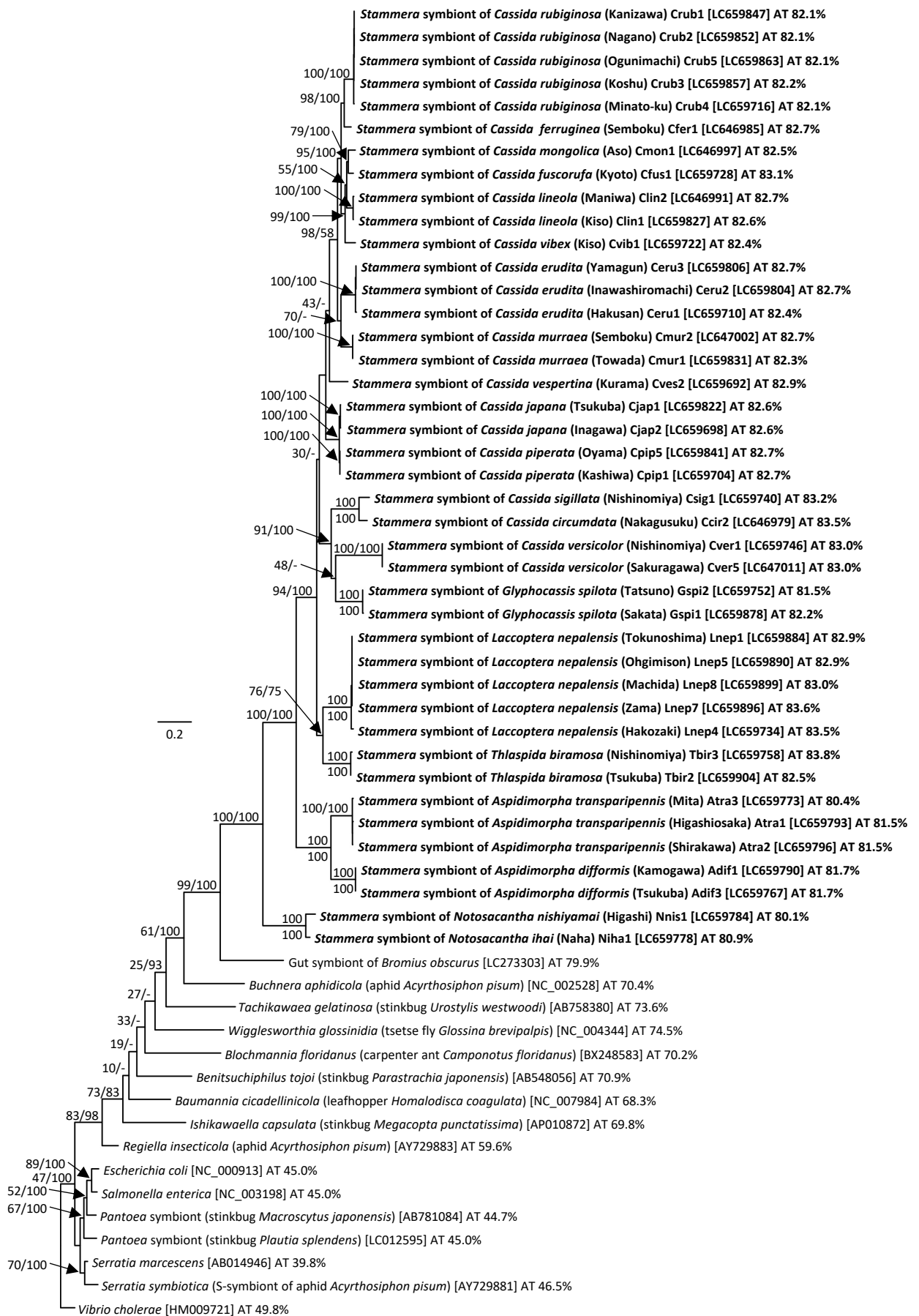

Fig. S2

Supplement: FIG S2 [file mbio.03691-21-sf002.pdf]

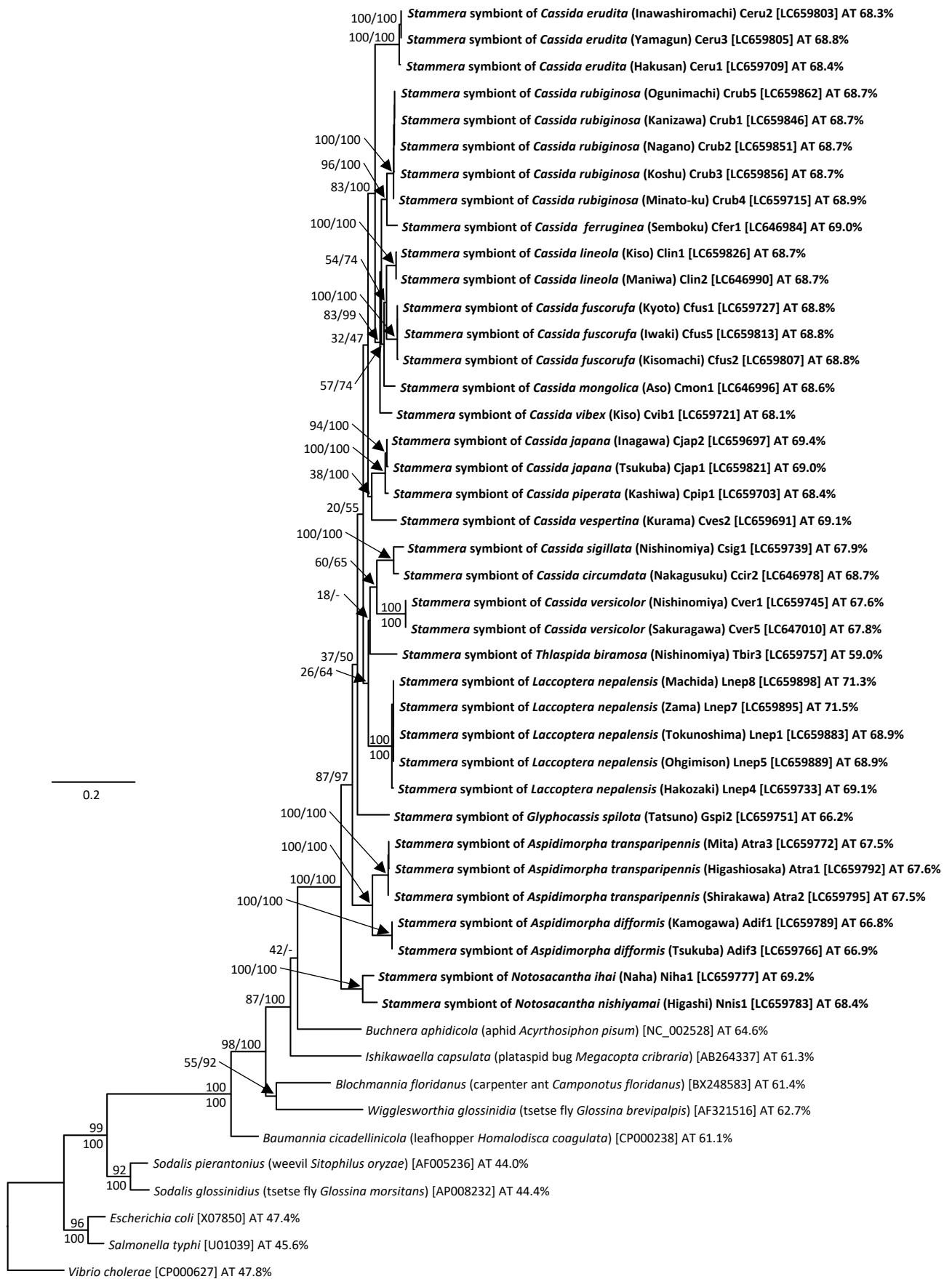

Fig. S3

Supplement: FIG S3 [file mbio.03691-21-sf003.pdf]

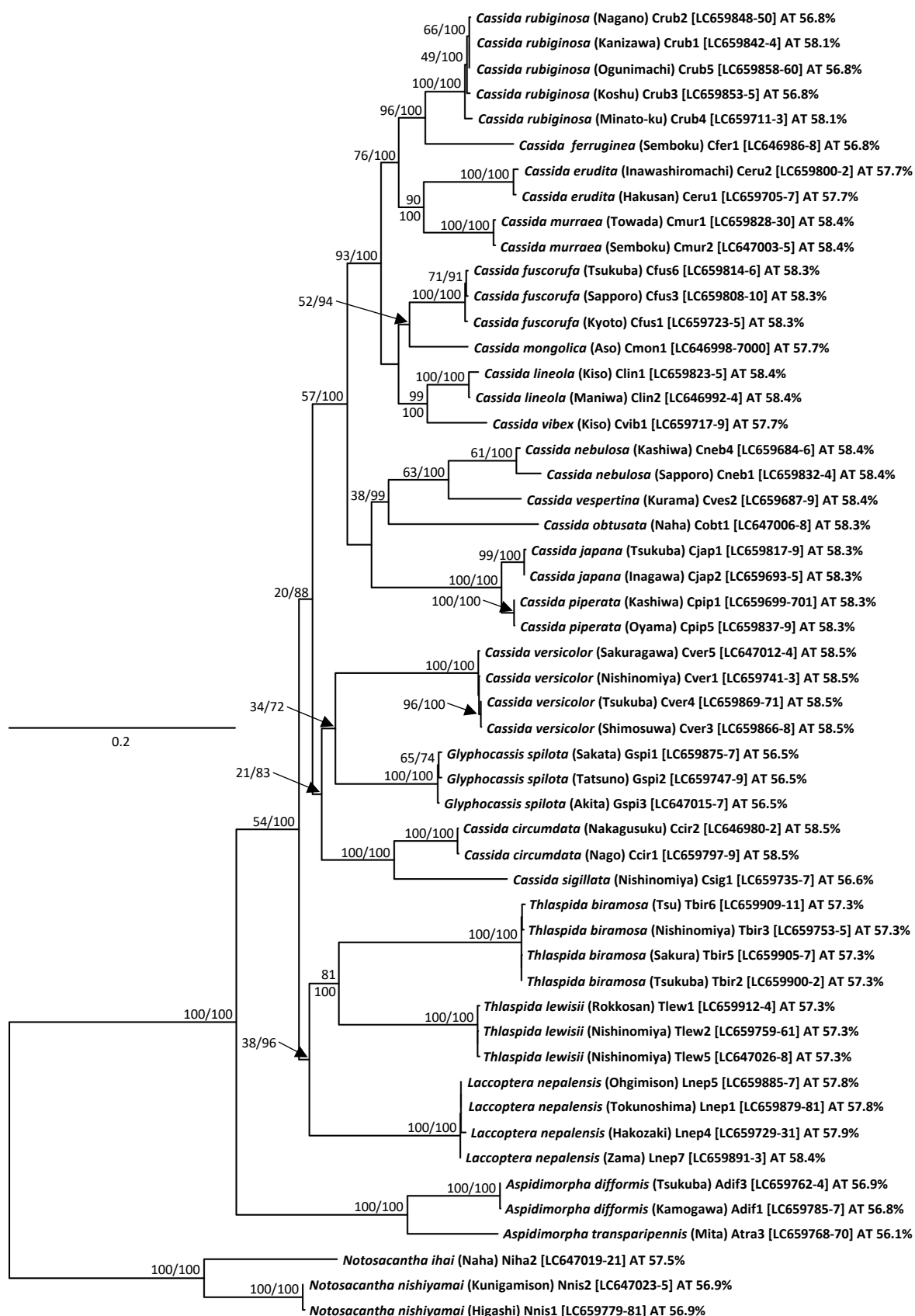

Fig. S4

Supplement: FIG S4 [file mbio.03691-21-sf004.pdf]

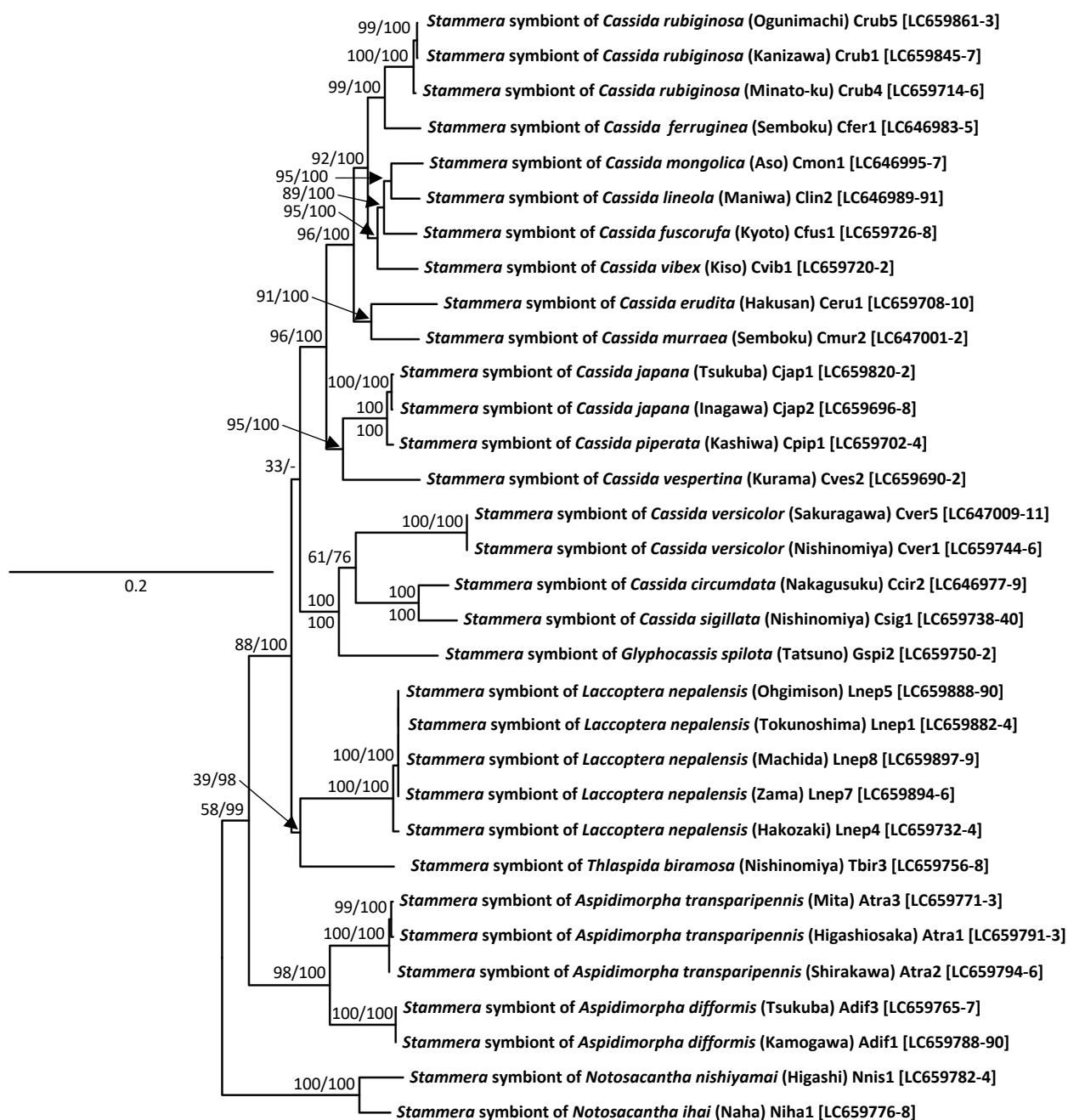

Fig. S5

Supplement: FIG S5 [file mbio.03691-21-sf005.pdf]

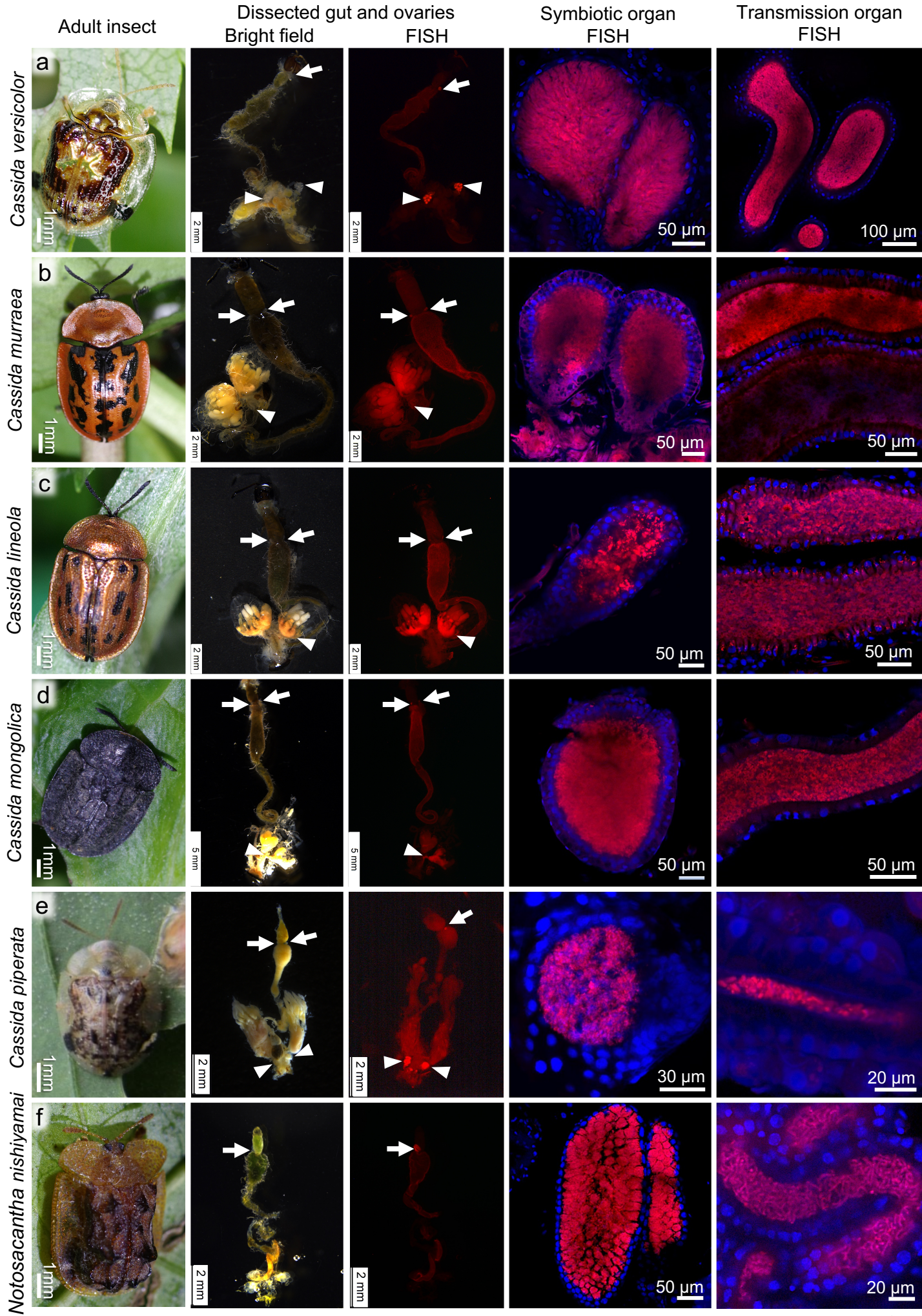

Fig. S6

Supplement: FIG S6 [file mbio.03691-21-sf006.pdf]
